# Structural and biochemical insights into the inhibition of *Mycobacterium tuberculosis* cyclic dinucleotide phosphodiesterase by a sulfur-modified cyclic dinucleotide analog

**DOI:** 10.1039/d6cb00006a

**Published:** 2026-04-14

**Authors:** Dagur Singh Hanuman, Singh Neeharika, Sinha Krishna Murari, Simpa K. Yeboah, Herman O. Sintim, Eerappa Rajakumara

**Affiliations:** a Macromolecular Structural Biology Laboratory, Department of Biotechnology, Indian Institute of Technology Hyderabad (IITH) Hyderabad Telangana-502285 India eraj@bt.iith.ac.in; b Amity Institute of Biotechnology, Amity University Haryana Haryana India; c James Tarpo Jr. and Margaret Tarpo Department of Chemistry 560 Oval Drive West Lafayette IN 47907 USA; d Department of Chemistry and Biochemistry, University of Notre Dame 305A McCourtney Hall Notre Dame IN 46556 USA hsintim@nd.edu

## Abstract

Cyclic dinucleotide (CDN) signaling plays a pivotal role in bacterial physiology and host–pathogen interactions. *Mycobacterium tuberculosis* (Mtb) releases a cyclic dinucleotide phosphodiesterase (CdnP) in the macrophages, which hydrolyzes Mtb-derived 3′3′-c-di-AMP and 3′3′-c-di-GMP, and a host-derived 2′3′-cGAMP STING agonist, to evade the host's innate immune response mediated by the STING protein. Therefore, by inhibiting CdnP released into host cells, the STING pathway can be potentiated, leading to improved bacterial clearance, which represents a potential novel approach for anti-tuberculosis (anti-TB) therapy. Here we report ES-2′3′-cAAMP, an analog of the host-derived STING agonist 2′3′-cGAMP, in which the phosphodiester bond is modified and the base is replaced, binds to CdnP with a micromolar binding affinity and competes with CdnP's substrates – 3′3′-c-di-AMP and 3′3′-c-di-GMP – binding to CdnP. Significantly, CdnP's phosphodiesterase catalytic activity is inhibited by ES-2′3′-cAAMP. Furthermore, the CdnP–ES-2′3′-cAAMP complex structure reported here is the first structure of the CdnP complexed with CDN, revealing the unique pose of ES-2′3′-cAAMP in the catalytic pocket of CdnP that is inaccessible to catalytic residues and Mn^2+^ ions for its hydrolysis, and in parallel blocks the binding of the natural substrates of CdnP that explains the structural basis of CdnP's catalytic activity inhibition by the inhibitor. Additionally, 2′3′-cGAMP in the STING receptor and ES-2′3′-cAAMP in the CdnP adopted an identical horseshoe conformation, suggesting that ES-2′3′-cAAMP, or an analogue thereof, can bind to and stimulate STING, thus acting as a synthetic STING agonist. These combined structural and biochemical findings provide new mechanistic insights into the inhibition of Mtb CdnP and offer a novel approach to host-directed anti-TB therapies that aim to enhance the host's own immune responses rather than directly killing the pathogen, which may help to mitigate the problem of antibiotic resistance.

## Abstract 2

ES-cAAMP binds to Mtb CdnP in a unique horseshoe conformation in the catalytic pocket (CG-site) away from the catalytic cation and residues, which are in the R-site. It competes with the substrate and inhibits CdnP's catalytic activity.

## Graphical abstract

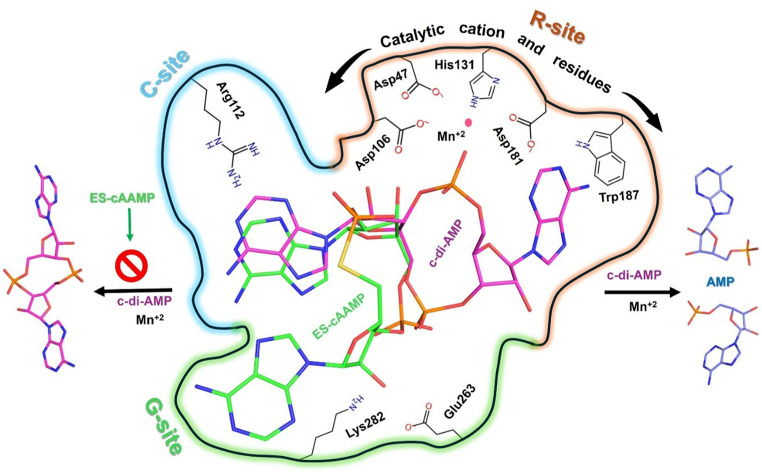

## Introduction

Cyclic dinucleotides (CDNs) are universal second messenger signaling molecules that regulate essential biological processes across bacteria, viruses, and eukaryotes.^1–7^ While they were originally discovered as regulators of bacterial physiology, they are now recognised as potent modulators of the mammalian immune system *via* the cyclic GMP/AMP synthase–stimulator of interferon genes (cGAS–STING) pathway, playing a central role in infectious diseases and cancer.^2,6,8–13^ CDN signalling in bacteria is ancient and emerged before the host's response to CDNs. The primary purpose of CDN secretion in bacteria remains to be elucidated, but in a few examples, secreted CDN can enhance bacterial fitness in response to extracellular CDNs. CDNs like 3′3′-c-di-GMP, 3′3′-c-di-AMP, and 3′3′-cyclic GMP–AMP (3′3′-cGAMP) regulate bacterial motility, biofilm formation, virulence, cell wall homeostasis, and stress survival.^13–16^ When bacteria invade host cells, they secrete CDNs (*e.g.*, *Listeria monocytogenes* secreting c-di-AMP), bypassing cGAS, which are sensed by the host receptor STING. This triggers a type I interferon (IFN) response, inducing autophagy to clear the pathogen.^17–19^

*Mycobacterium tuberculosis* (Mtb) and cancer both manipulate the host's STING immune pathway, often by disrupting cyclic dinucleotide signaling, using bacterial and host phosphodiesterases (PDEs) to break down immune-activating molecules.^7,10,11,20^ Mtb, *Streptococcus pneumoniae* and other infectious bacteria release cyclic dinucleotides (CDNs) like 3′3′-c-di-AMP and 3′3′-c-di-GMP, which activate the host STING pathway (boosting interferon), but also secretes the cyclic dinucleotide phosphodiesterase (CdnP, also known as CnpB) to hydrolyze both bacterial 3′3′-c-di-AMP and 3′3′-c-di-GMP, and the host's endogenous 2′3′-cGAMP (STING agonist), effectively removing the “alarm” molecules and dampening the immune response for survival that would otherwise activate STING and trigger a type I interferon (IFN) response.^7,15,20–25^ PDE inhibitors could augment anti-TB drugs by restoring immune function *via* the inhibition of the hydrolysis of CDNs by Mtb CdnP.^26^

During Mtb infection, CdnP is released into the cytosol of host macrophages, probably *via* autolysis, and the CdnP degrades the host-derived 2′3′-cGAMP, a natural STING agonist, and circumvents the host's surveillance system.^11,20^ Inhibiting Mtb's CdnP enzyme is a promising strategy to combat tuberculosis (TB) because Mtb CdnP helps the bacteria evade the host immune system, and blocking it enables the body's immune defence to function more effectively, leading to an enhanced anti-TB response.^11,26^ Host's endogenous 2′3′-cGAMP is less susceptible to hydrolysis by Mtb CdnP because it has a non-canonical 2′–5′ phosphodiester linkage in addition to a canonical 3′–5′ phosphodiester linkage compared to highly susceptible bacterial 3′3′-c-di-AMP and 3′3′-c-di-GMP, which have two canonical 3′–5′ phosphodiester linkages.^11,21^

We have selected a chemically synthesized *endo-S*-2′3′-cAAMP (hereafter abbreviated as ES-2′3′-cAAMP)^27^ to probe its inhibition potency against Mtb CdnP because (A) it may bind to and inhibit CdnP because the 2′–5′ phosphodiester bond of ES-2′3′-cAAMP is modified by the substitution of oxygen to sulfur, so that it might be resistant to hydrolysis by CdnP and host ENPP1 (ectonucleotide pyrophosphatase/phosphodiesterase 1), (B) the *endo-S*-2′3′-cAAMP has phosphodiester linkages (dictating the conformation of CDN) similar to 2′3′-cGAMP, suggesting that it may bind to and activate STING and be less susceptible to hydrolysis by Mtb CdnP, and (C) the guanine base is replaced by adenine to mimic Mtb's 3′3′-c-di-AMP so that it may bind to Mtb CdnP with higher affinity compared to 2′3′-cGAMP.^27^

To test the above assumptions that ES-2′3′-cAAMP can bind to and inhibit Mtb's CdnP, we carried out isothermal titration calorimetry (ITC) binding and competition binding studies, which revealed that ES-2′3′-cAAMP not only binds to CdnP but also competes with natural substrates binding to CdnP. Furthermore, our studies showed that ES-2′3′-cAAMP inhibits the phosphodiesterase catalytic activity of Mtb CdnP. The CdnP–ES-2′3′-cAAMP complex structure that we resolved is the first structure of the Mtb CdnP complexed with CDN, revealing that ES-2′3′-cAAMP adopted the horseshoe-shaped conformation (shortened to horseshoe conformation) and is located farther from the catalytic Mn^2+^ in the catalytic pocket of Mtb's CdnP, explaining the structural basis of the inhibition of Mtb CdnP catalytic activity by ES-2′3′-cAAMP. Additionally, 2′3′-cGAMP in the STING receptor and ES-2′3′-cAAMP in the Mtb CdnP adopted an identical horseshoe conformation, suggesting that ES-2′3′-cAAMP can bind to and stimulate STING, thus acting as a synthetic STING agonist.

## Results

### ITC binding studies of ES-2′3′-cAAMP and substrates to CdnP

**Fig. 1:**
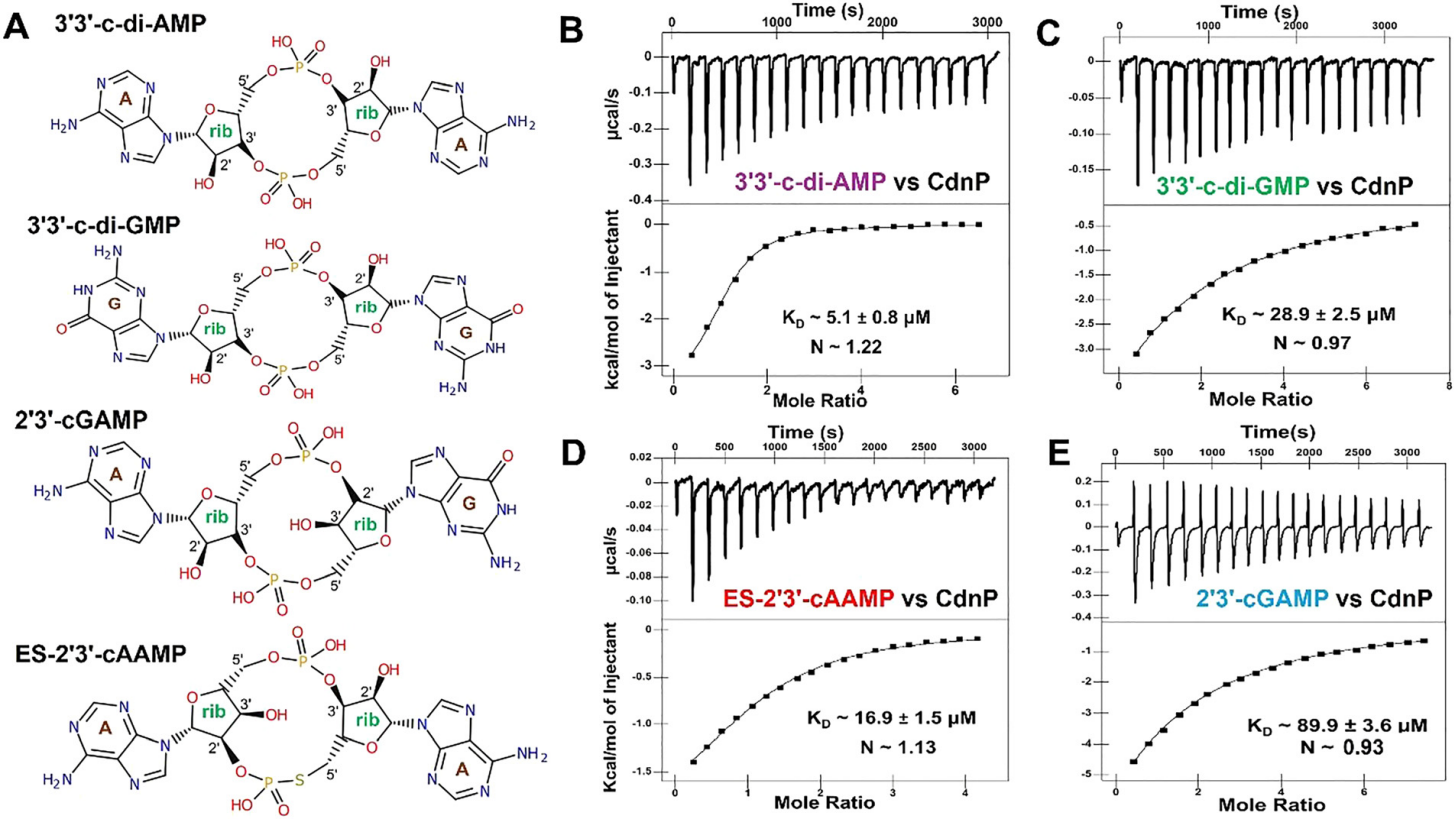
Chemical structures of cyclic dinucleotides (CDNs) and their ITC binding studies with Mtb CdnP. (A) Schematic representation of the 2D structures of 3′3′-c-di-AMP, 3′3′-c-di-GMP, 2′3′-cGAMP, and ES-2′3′-cAAMP. Substrates of CdnP 3′3′-c-di-AMP and 3′3′-c-di-GMP have two canonical 3′–5′ phosphodiester bonds. In contrast, 2′,3′-cGAMP and ES-2′3′-cAAMP have a canonical 3′–5′ and a non-canonical 2′–5′ bond. In ES-2′3′-cAAMP, the bridging oxygen and guanine are replaced by a sulfur atom and an adenine base, respectively. ITC thermograms of CdnP binding to (B) 3′3′-c-di-AMP, (C) 3′3′-c-di-GMP, (D) ES-2′3′-cAAMP, and (E) 2′3′-cGAMP.

Given that ES-2′3′-cAAMP mimics c-di-AMP since it contains two adenine bases (Fig. 1A), we carried out ITC binding studies to compare the binding affinity of ES-2′3′-cAAMP and CDN substrates to Mtb CdnP. Recombinant Mtb CdnP (Fig. S1A) binds to 3′3′-c-di-AMP and 3′3′-c-di-GMP with *K*_D_ ≈ 5.1 and 28.9 µM affinity, respectively (Fig. 1B and C). The Mtb CdnP binds to *endo-S*-2′3′-c-AAMP (abbreviated as ES-2′3′-cAAMP) with *K*_D_ ≈ 16.9 µM affinity (Fig. 1D), which is 3-fold less and 2-fold higher than the binding affinity of Mtb CdnP to 3′3′-c-di-AMP and 3′3′-c-di-GMP, respectively (Fig. 1B–D). The Mtb CdnP binds to ES-2′3′-cAAMP with ∼5.3-fold higher affinity than binding to 2′3′-cGAMP (Fig. 1D and E).^11^ Thermodynamic parameters, including Gibbs free energy (Δ*G*), enthalpy (Δ*H*) and entropy (Δ*S*), are provided in Table 1.

**Table 1:** Thermodynamic parameters obtained from ITC studies of CdnP binding to different CDNs

CDN	*K*_D_ (M)	Δ*H* (kcal mol^−1^)	Δ*S* (cal mol^−1^ K^−1^)	Δ*G* (kcal mol^−1^)
c-di-AMP	5.1 × 10^−6^	−3.59	11.57	−6.97
c-di-GMP	28.9 × 10^−6^	−22.95	−57.59	−6.08
ES-2′3′cAAMP	16.9 × 10^−6^	−2.25	14.27	−6.51
2′3′cGAMP	89.9 × 10^−6^	−25	−65.33	−5.52

### ES-2′3′-cAAMP weakens the binding affinity of substrate CDNs to Mtb CdnP

**Fig. 2:**
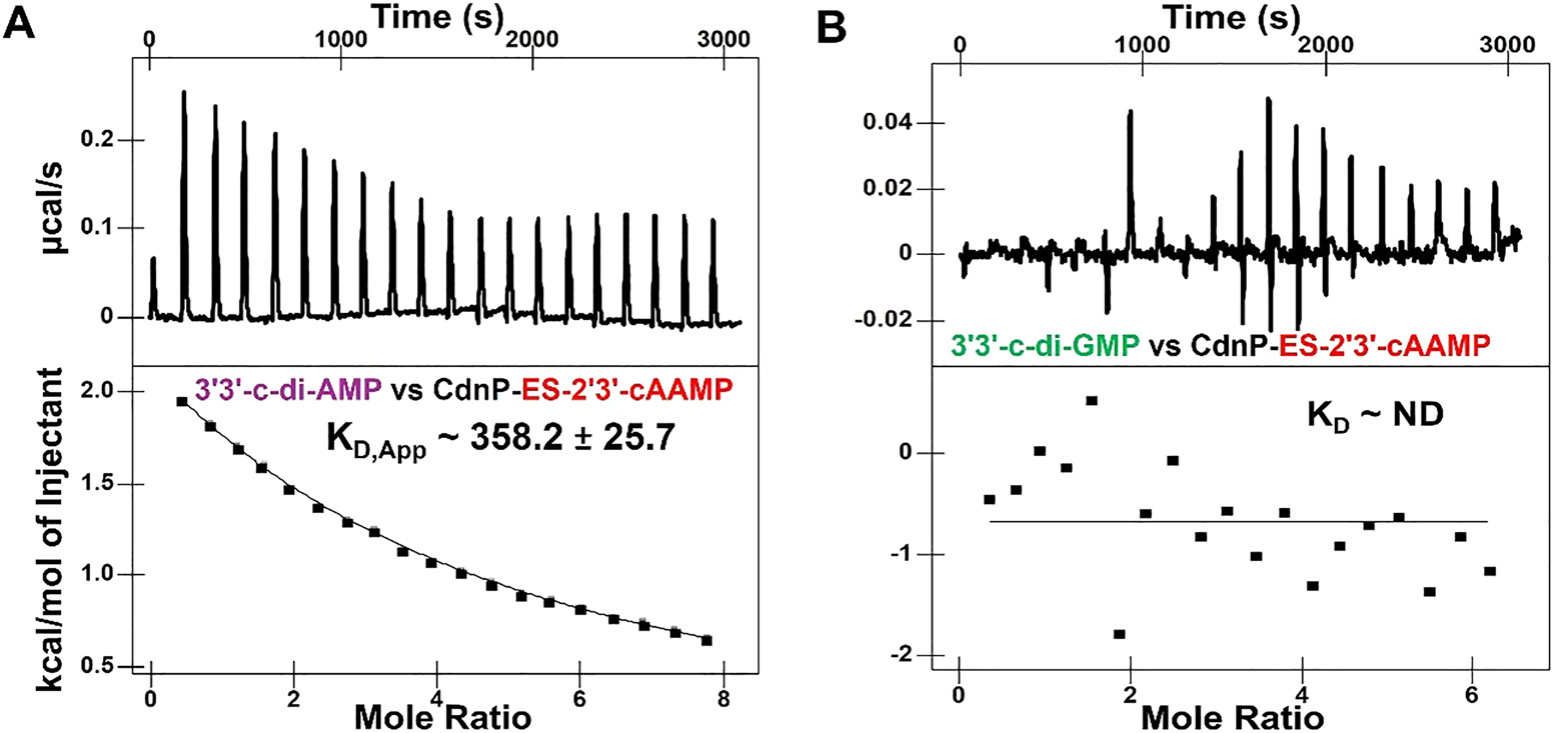
ITC competition binding analysis of CdnP. ITC thermograms of (A) 3′3′-c-di-AMP binding to a 1 : 1 CdnP–ES-2′3′-cAAMP complex and (B) 3′3′-c-di-GMP binding to a 1 : 1 CdnP–ES-2′3′-cAAMP complex.

Having established that both the natural substrates and ES-2′3′-cAAMP bind to Mtb CdnP (Fig. 1B–E), we investigated whether the binding of ES-2′3′-cAAMP to Mtb CdnP reduces the affinity of the natural substrates to Mtb CdnP. We measured the binding affinity of 3′3′-c-di-AMP or 3′3′-c-di-GMP to the CdnP–ES-2′3′-cAAMP complex with a 1 : 1 molar ratio. The binding affinity (*K*_D,cAMP/cAAMP_ ≈ 358.2 µM) of c-di-AMP to the CdnP–ES-2′3′-cAAMP complex is ∼70-fold weaker than its affinity to apo-CdnP (Fig. 2A). On the other hand, c-di-GMP did not show any affinity towards the same binary CdnP–ES-2′3′-cAAMP complex (Fig. 2B). Together, these binding studies demonstrate that ES-2′3′-cAAMP effectively competes for the natural CDN substrates’ recognition by Mtb's CdnP.

### ES-2′3′-cAAMP inhibits the phosphodiesterase activity of Mtb CdnP

**Fig. 3:**
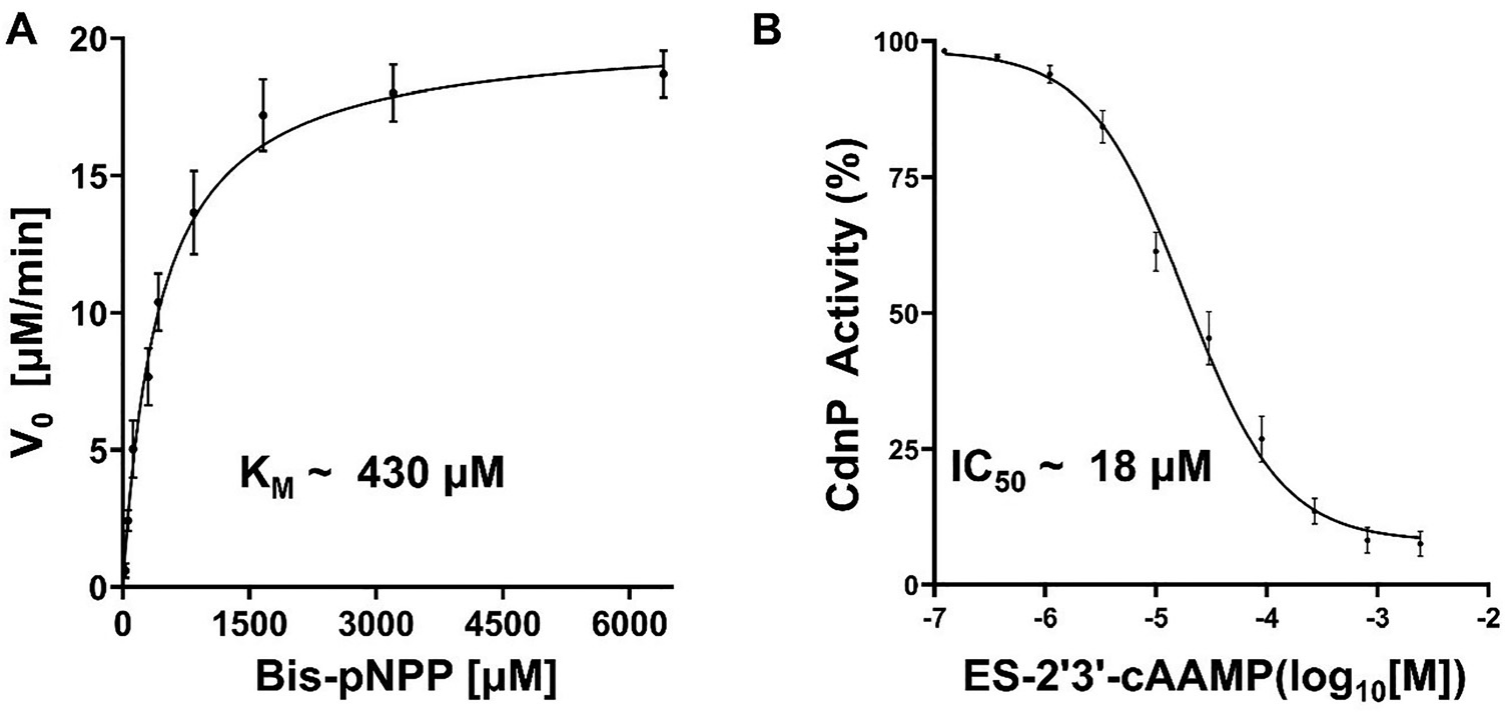
Kinetic and inhibition analysis of the phosphodiesterase catalytic activity of Mtb CdnP by ES-2′3′-cAAMP. (A) Michaelis–Menten kinetics of bis-pNPP catalysis by the CdnP. (B) CdnP catalytic activity inhibition by ES-2′3′-cAAMP. The IC_50_ measurements were conducted by varying ES-2′3′-cAAMP concentrations in the presence of 4.5 mM substrate incubated for 30 minutes. All data are mean ± SEM (*n* = 3).

Given that ES-2′3′-cAAMP weakens the natural CDN substrates’ recognition by the CdnP, we assume that ES-2′3′-cAAMP may inhibit the catalytic activity of CdnP. We carried out the enzyme inhibition assay to find out how well ES-2′3′-cAAMP may inhibit the catalytic activity of the Mtb CdnP. The non-CDN substrate bis-pNPP was utilized to measure the catalytic activity of Mtb CdnP. Kinetic parameters were determined by measuring the rate of *p*-nitrophenol (*p*-NP) release in the presence of increasing bis-pNPP concentrations.^15^ The Michaelis–Menten analysis yielded a *K*_M_ of approximately 430 µM, a *V*_max_ of 20 µM min^−1^, and a *k*_cat_ of 13.5 s^−1^ (Fig. 3A). Inhibition studies have shown that ES-2′3′-cAAMP inhibited Mtb CdnP with an IC_50_ of ∼18 µM and a *K*_i_ of ∼1.3 µM (Fig. 3B), indicating that ES-2′3′-cAAMP may act as a moderate inhibitor of Mtb CdnP's catalytic activity.

### Co-crystal structure of the CdnP–*endo-S*-2′3′-cAAMP complex

**Fig. 4:**
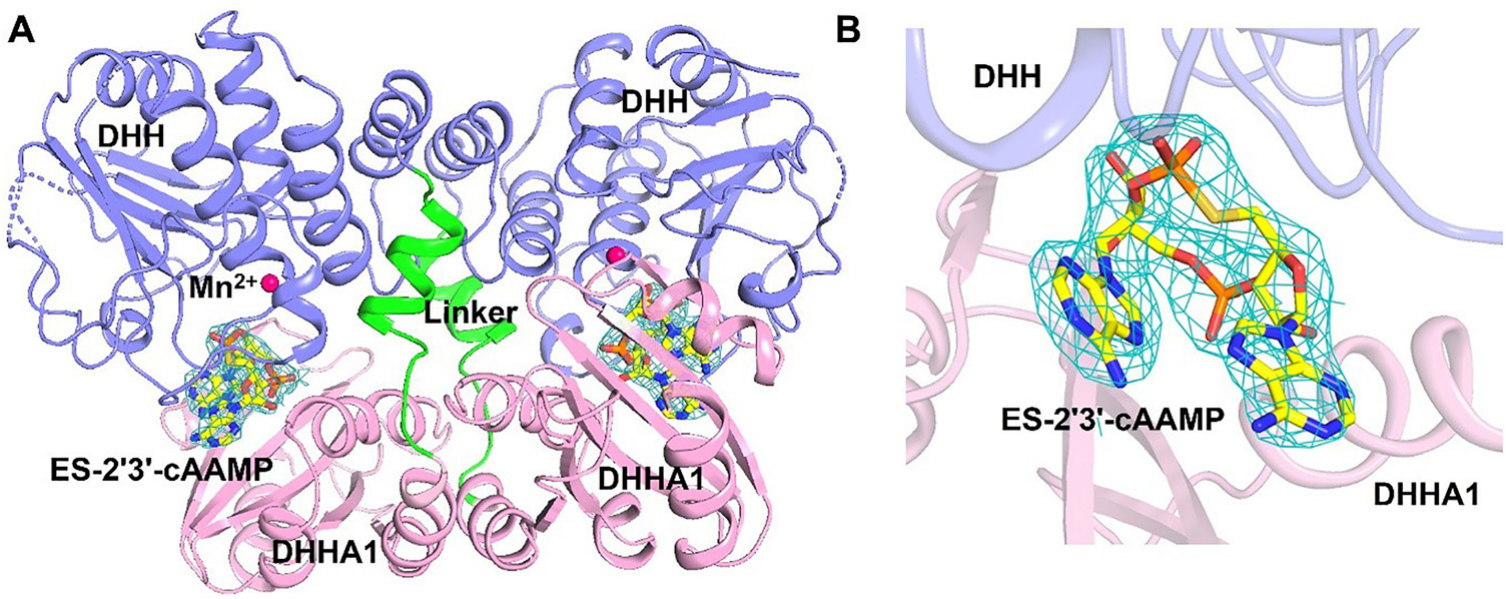
Co-crystal structure of the CdnP–ES-2′3′-cAAMP complex. (A) Structure of the biological dimer assembly and ES-2′3′-cAAMP bound to each subunit of the dimer. Individual subunits are color-coded in slate blue and light pink in a cartoon representation, whereas the bound ES-2′3′-cAAMP is represented as a yellow stick. The variable linker connects the two domains, which is shown in green. (B) ES-2′3′-cAAMP is bound in the cleft formed between the DHHA1 (colored in light pink) and DHH (colored in slate blue) domains. ES-2′3′-cAAMP is modeled in the 2Fo–Fc electron density map (contoured at 1.5*σ*).

The structure of the Mtb CdnP complexed with a natural or synthetic CDN has not been reported to date. Binding and competition studies have shown that ES-2′3′-cAAMP (*endo-S*-2′3′-cAAMP) may bind to Mtb CdnP in the catalytic pocket, where 3′3′-c-di-AMP and 3′3′-c-di-GMP bind. To understand the structural basis of the inhibition of the CdnP's catalytic activity by ES-2′3′-cAAMP, a non-hydrolysable analog of cGAMP, and the mode of recognition of a CDN by the Mtb CdnP, we crystallized the CdnP–ES-2′3′-cAAMP binary complex (Fig. S1B), then resolved the complex structure by X-ray crystallography. The complex crystallized in the *I*2_1_2_1_2_1_ space group and diffracted to a resolution of 2.96 Å. The structure was determined by molecular replacement using the apo CdnP structure (PDB ID: 5CET) as the search model. Data processing and refinement statistics are summarized in Table 2. The asymmetric unit (ASU) contains a total of three monomers (Fig. 4A and Fig. S2).

Crystallographic data collection and refinement statistics for the Mtb CdnP–ES-2′3′-cAAMP complex

**Table:** 

Collection	
Wavelength (Å)	1.542
Resolution range (Å)	24.77–2.96 (3.12–2.96)
Space group	*I*2_1_2_1_2_1_

**Table:** 

Unit cell	
*a*, *b*, *c* (Å)	79.01, 149.09, 167.03
*α* = *β* = *γ* (°)	90
Multiplicity	5.1 (5.1)
Completeness (%)	99.62 (99.86)
Mean *I*/sigma(*I*)	4.7 (1.0)
*R*-merge	0.32 (1.2)
*R*-pim	0.17 (0.86)
CC1/2	0.96 (0.72)

**Table:** 

Refinement	
Reflections used	20 865 (2940)
Reflections (*R*-free)	1035 (152)
*R*_work_/*R*_free_ (%)	22.68/27.86
Non-hydrogen atoms	6912
Macromolecules	6773
Ligands	139

**Table:** 

RMSD	
Bond length (Å)	0.008
Bond angle (°)	1.56
Ramachandran favored (%)	94.97
Ramachandran outliers (%)	0.22
Ramachandran allowed (%)	4.81
Average *B*-factor (Å^2^)	49.44
Macromolecules	49.36
Ligands	53.20
PDB ID	9WBU

**Table 3:** Details of different conformations of cyclic dinucleotides (CDNs), along with their corresponding binding proteins or receptors and associated PDB codes, used in the study

Name of CDN	PDB ID	CDN conformation	Name of protein binding to CDN
3′3′-c-Di-AMP	8AD6	Horseshoe	CBS domain-containing protein
3′3′-c-Di-AMP	5XSN	Extended	GGDEF domain protein containing phosphodiesterase (GdpP)
3′3′-c-Di-GMP	5XT3	Extended	GGDEF domain protein containing phosphodiesterase (GdpP)
3′3′-c-Di-GMP	3Q3Z	Horseshoe	c-Di-GMP-II riboswitch
3′3′-c-GAMP	6AEL	Extended	ENPP1 complexed with 3′3′-cGAMP
3′3′-c-GAMP	8H2J	Horseshoe	Acb2 complexed with 3′,3′-cGAMP
2′3′-c-GAMP	5GRM	Horseshoe	Stimulator of interferon genes (STING) protein
*endo-S*-2′3′-c-AAMP	9WBU	Horseshoe	Mtb cyclic dinucleotide phosphodiesterase (CdnP)^a^

a Current study.

Among these, two monomers form the biological dimer, while the dimeric partner of the third monomer is generated through crystallographic symmetry expansion (Fig. S2). Each monomer of a dimer contains two domains: DHH (residues 15–202) and DHHA1 (residues 220–336). These two domains are connected by a linker (residues 203–219), which is reported to determine the substrate specificity (Fig. 4A).^21,28^ We observed the well-defined Fo–Fc difference map's electron density (at 2.5*σ*) within the cleft at the interface of the two domains, where the substrates bind. ES-2′3′-cAAMP was modeled into this unaccounted density. The structure was subsequently refined, and there were no positive or negative densities in the Fo–Fc difference map surrounding ES-2′3′-cAAMP (Fig. 4B), suggesting that the unaccounted electron density is that of ES-2′3′-cAAMP. The *B*-factors of the ligand were comparable to those of the surrounding protein residues, supporting the presence of ES-2′3′-cAAMP in the co-crystal structure at full occupancy.

**Fig. 5:**
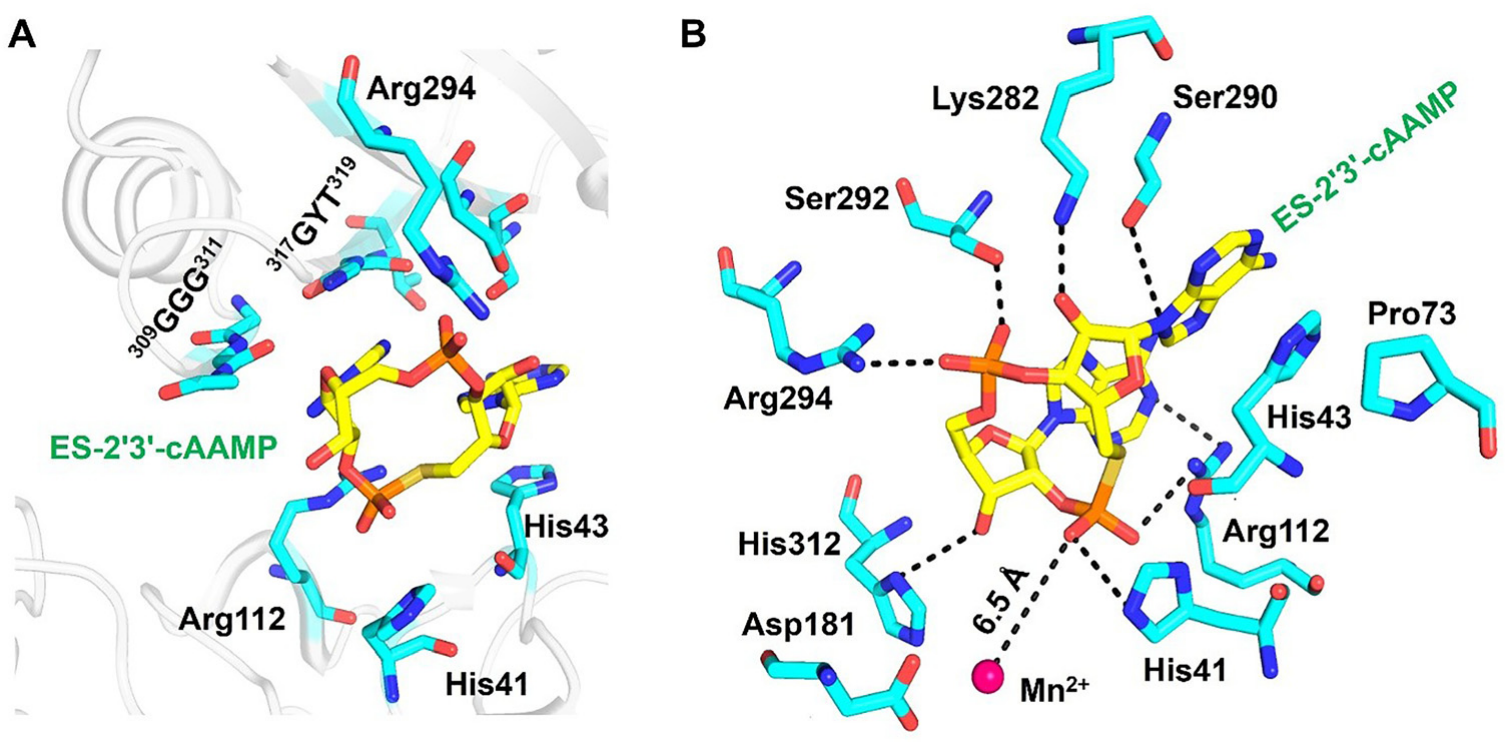
Molecular interactions between Mtb CdnP and ES-2′3′-cAAMP. (A) Stacking interactions of 4–5 layers, due to which ES-2′3′-cAAMP is trapped in the substrate binding pocket. (B) Hydrogen bond interactions with the distance of Mn^2+^ are indicated by dashed lines. ES-2′3′-cAAMP is shown as sticks in yellow and the interacting residues of Mtb CdnP in cyan. The protein backbone is shown as a cartoon.

The previously reported apo structure of Mtb CdnP contains two Mn^2+^ ions at the catalytic pocket. However, our co-crystal structure reveals only one Mn^2+^ ion (Fig. S3), coordinated by Asp47, Asp106, and Asp181 residues.^21^ The Mn^2+^ ion adopts a tetrahedral geometry (Fig. S3). The Mn^2+^ ion is ∼6.5 Å away from the nearest phosphorus of ES-2′3′-cAAMP, indicating that the ligand is not positioned for catalysis (Fig. 5). ES-2′3′-cAAMP adopts a horseshoe conformation (Fig. 5A) and is stabilized at the interdomain cleft through parallel-displaced intra- and inter-molecular stacking interactions and intermolecular hydrogen bonding interactions. Both adenine bases establish intermolecular stacking interactions (Fig. 5A). Arg112 and the peptide bonds of residues 309–311 and 317–319 establish stacking interactions with one of the adenines below and above, respectively. Arg112 is not only co-planar to another adenine base but further stacks with His43, supported by Pro73 and the peptide plane of residues 72–73. Ser290 also forms a hydrogen bond with this adenine base from the top (Fig. 5B).

Together, these interactions form 4–5 layers of intra- and intermolecular stacking that unambiguously anchor ES-2′3′-cAAMP. Arg112 and Arg294/Ser292 form hydrogen bonds with the 2′–5′ and 3′–5′ phosphate groups, respectively (Fig. 5B). His41 also forms a hydrogen bond with the 2′–5′ phosphate group (Fig. 5B). The 2′-OH and 3′-OH groups of ribose sugars form hydrogen bonds with Lys282 and His312, respectively (Fig. 5B). Arg112 also shows a cation–π interaction with one of the adenine bases. These combined interactions from the base, sugar, and phosphate moieties contribute to the specific molecular recognition that validates the observed binding affinity between Mtb CdnP and ES-2′3′-cAAMP.

### Structural basis of inhibition of the catalytic activity of the Mtb CdnP by ES-2′,3′-cAAMP

**Fig. 6:**
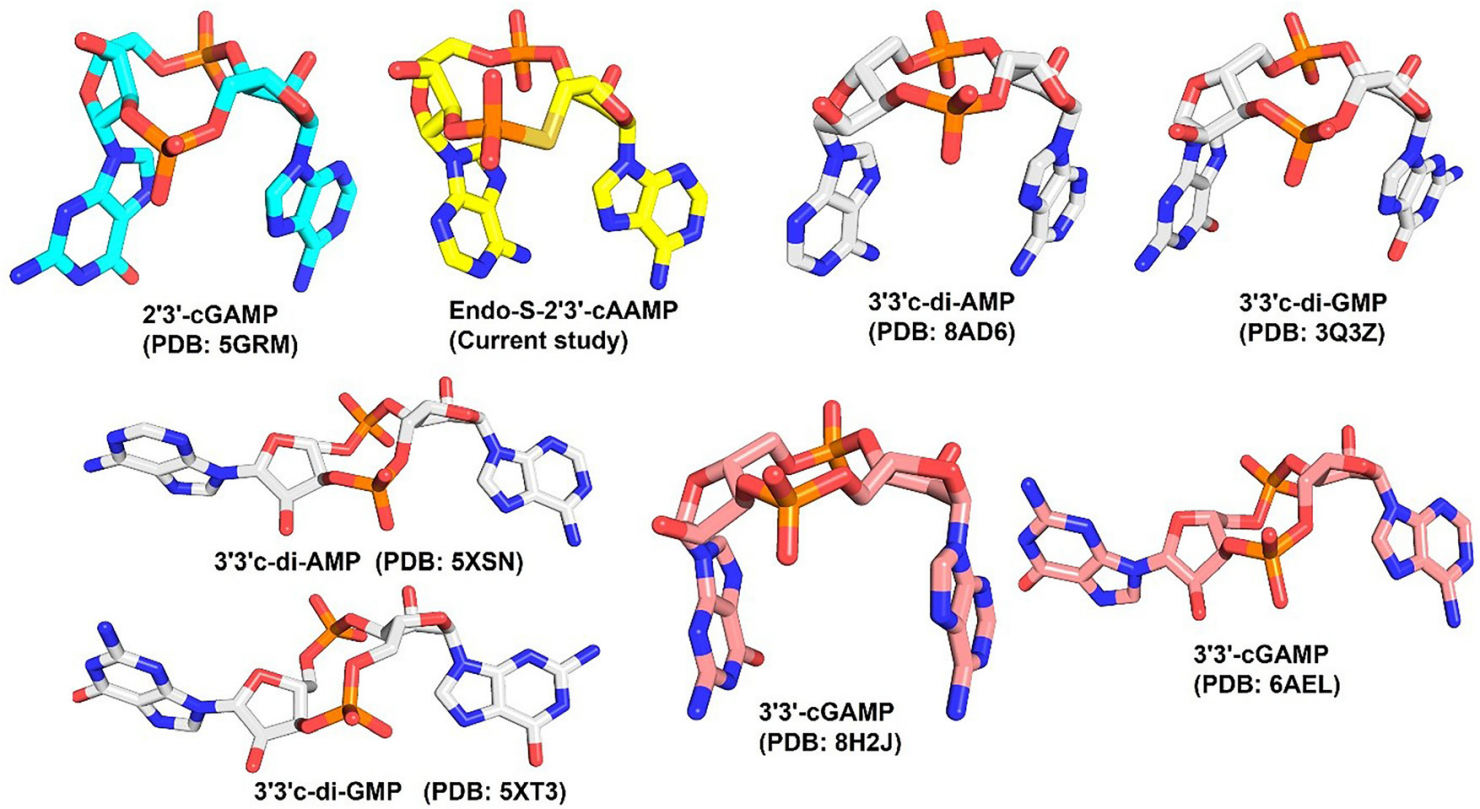
Structural conformations of naturally occurring CDNs. ES-2′3′-cAAMP and 2′3′-cGAMP adopt horseshoe conformation, whereas 3′3′-c-di-AMP, 3′3′-c-di-GMP, and 3′3′-cGAMP adopt both horseshoe and extended conformations. Details of the PDB codes used in this figure are provided in Table 3 for clarity.

**Fig. 7:**
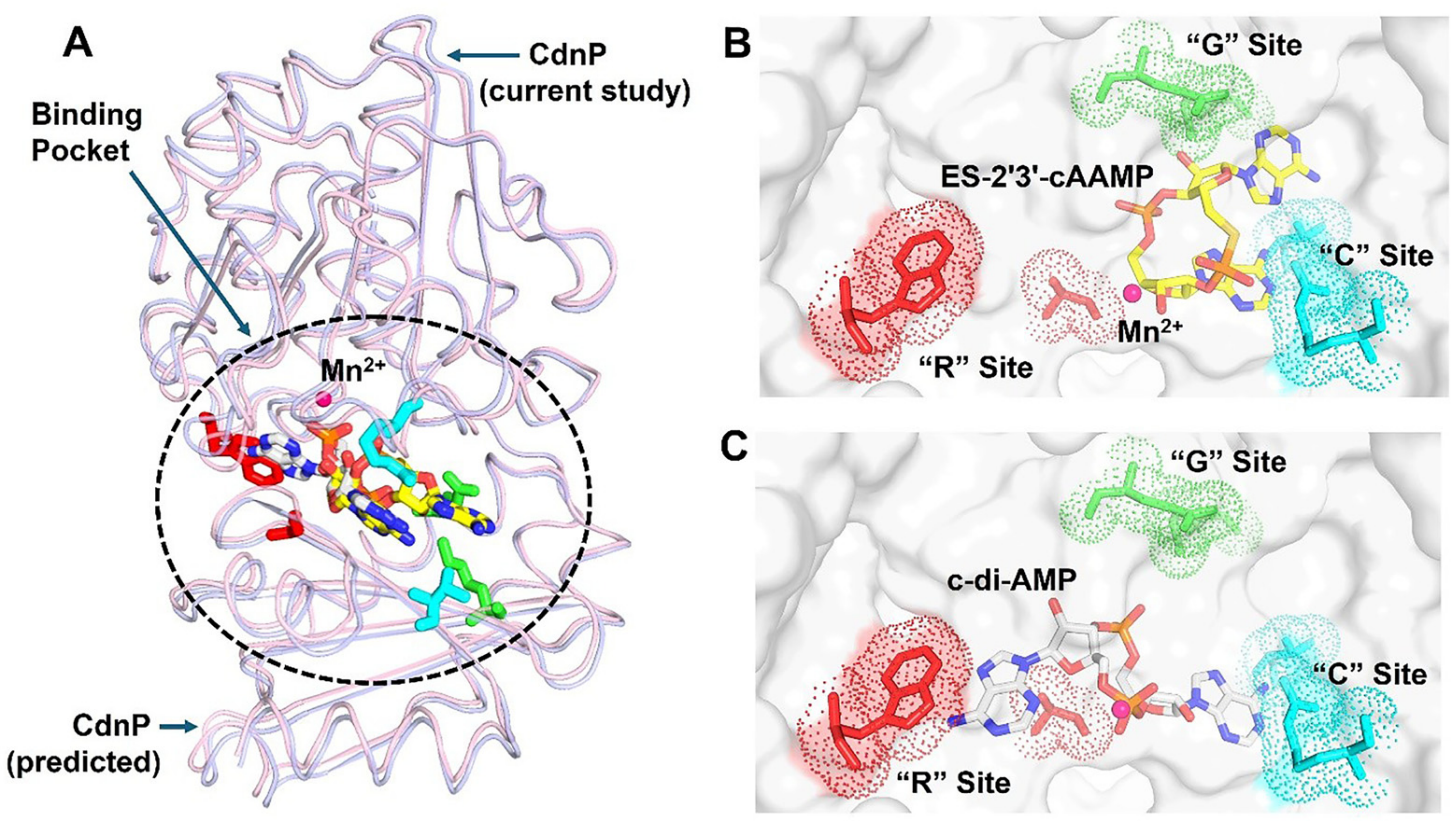
Substrate binding pockets of CdnP and the conformations of the inhibitor ES-2′3′-cAAMP and the substrate 3′3′-c-di-AMP in the catalytic pocket. (A) Superposition of the crystal structure of the CdnP–ES-2′3′-cAAMP complex on the AlphaFold3-predicted structure of the CdnP–3′3′-c-di-AMP complex. Mtb CdnP in the co-crystal structure and the AlphaFold3-predicted structure is shown as a cartoon in blue and pink colors, respectively. The binding pocket of Mtb CdnP is marked with a black circle. (B) The crystal structure of the CdnP–ES-2′3′-cAAMP complex. ES-2′3′-cAAMP adopted a horseshoe conformation and occupied the GC site, while the R-site, which hosts the catalytic Mn^2+^ and aspartate, remained empty. (C) Predicted structure of the CdnP–3′3′-c-di-AMP complex. The 3′3′-c-di-AMP adopted an extended conformation and occupied the RC site, while the G-site remained empty. Subsites ‘C’ (Arg112 and Thr319), R (Trp187 and Ala315), and ‘G’ (Glu263 and Lys282) are indicated in cyan, red, and green colors, respectively. The ES-2′,3′-cAAMP and 3′3′-c-di-AMP are displayed in yellow and white stick representation, respectively, and the protein is shown as a surface in (B) and (C).

The catalytic efficiency of Mtb CdnP to hydrolyze the natural STING agonist 2′3′-cGAMP is thousand-fold lower than that of its natural substrates 3′3′-c-di-AMP and 3′3′-c-di-GMP.^11^ Furthermore, our current study has revealed that ES-2′3′-cAAMP inhibits the catalytic activity of CdnP. The commonality of 2′3′-cGAMP and ES-2′3′-cAAMP is that they both have a non-canonical 2′–5′ phosphodiester bond, which is absent in the above natural substrates (Fig. 1A). In the case of 3′3′-c-di-AMP and ES-2′3′-cAAMP, they both have adenine bases. So, ES-2′3′-cAAMP is an analog of both 2′3′-cGAMP and 3′3′-c-di-AMP. Our comparative structural analyses revealed that 2′3′-cGAMP and ES-2′3′-cAAMP adopted a horseshoe-shaped conformation compared to the extended conformation of 3′3′-c-di-AMP and 3′3′-c-di-GMP in the different receptors or enzymes (Fig. 6 and S4A). Phosphodiesterases have three interconnected sub-sites, R, C, and G, within the catalytic pocket (Fig. 7A and B).^21,28^ The R-site hosts the catalytic residue Asp181, both Mn^2+^ ions, and the catalytic water. CDN nucleotides bind to either the RC-site or the GC-site. ES-2′3′-cAAMP adopts a horseshoe conformation, and with this conformation, ES-2′3′-cAAMP is bound in the GC-site (Fig. 5B and 7B), thereby adopting a catalytically incompetent pose.^21,28^ In the case of GdpP, which is a close structural homolog of Mtb CdnP, 3′3′-c-di-AMP, 3′3′-c-di-GMP and 5′pApA are bound in the extended conformations in the catalytic pocket of GdpP,^28^ while ES-2′3′-cAAMP adopts a horseshoe-shaped conformation in the Mtb CdnP's catalytic pocket (Fig. S4A and B). Both 3′3′-c-di-AMP and 5′pApA occupy the GC site in GdpP (a close structural homolog of CdnP) (Fig. S4B). The R-site of GdpP is not sufficiently spacious to accommodate a nucleoside moiety, which likely explains its inability to sequentially hydrolyze c-di-AMP into two molecules of AMP.^28^

Due to the lack of reports on Mtb CdnP's structure complexed with CDN substrates or analogs, we predicted the structure of the CdnP–3′3′-c-di-AMP complex using AlphaFold3. In contrast, the structure of the CdnP–3′,3′-c-di-AMP complex revealed that 3′3′-c-di-AMP, with two canonical 3′–5′ phosphodiester bonds, occupies the RC site with an extended conformation (Fig. 7A and C). The extended conformation of 3′3′-c-di-AMP is complementary to the geometry of the RC-site. In this pose, the scissile phosphodiester bonds are easily accessible to the catalytic components – Asp181, both Mn^2+^ ions, and the catalytic water – in the phosphodiesterase Mtb CdnP and TmPDE.^21,29^ This observation suggested that 3′3′-c-di-AMP could be recognized by Mtb CdnP in the RC-site with an extended conformation for its sequential degradation to 5′-pApA, then to two AMP molecules through a flipping mechanism.^21,28,29^

Due to the binding of the CdnP–ES-2′3′-cAAMP complex in the GC-site (Fig. 7B), the distance between the phosphodiester bond of CdnP–ES-2′3′-cAAMP is 8.5 Å and 9.8 Å from the carboxyl side chain of Asp181, and 6.5 Å and 9.5 Å from the catalytic Mn^2+^ ion, respectively (Fig. 5B). Several other studies have shown that two Mn^2+^ ions are required for the catalysis,^11,21,28,29^ but in the current study the CdnP–ES-2′3′-cAAMP co-structure has only one Mn^2+^ ion, which is far from the phosphodiester bond of CdnP–ES-2′,3′-cAAMP (Fig. 5B). Also, Asp181 plays an unequivocal role in catalysis by coordinating with two Mn^2+^ ions and activating a catalytic water molecule to generate nucleophilic attack for phosphodiester bond cleavage.^11,21,28,29^ However, in the CdnP–ES-2′3′-cAAMP co-structure, the phosphorus atoms are located too far from Asp181 and the Mn^2+^ ions for catalysis to occur. Additionally, the conformation and orientation of CdnP–ES-2′3′-cAAMP within the catalytic pocket may have prevented another Mn^2+^ from binding to the catalytic pocket, thereby causing the rearrangement of the catalytic residues' coordination. As discussed above, the binding pose of CdnP–ES-2′3′-cAAMP and its orientation and conformation likely impair CdnP's catalytic activity.

## Discussion

This study has originated due to two reasons: (A) both host- and *Mycobacterium*-derived 2′3′-cGAMP and 3′3′-c-di-AMP, respectively, induce effective immune responses against Mtb infection, and overproduction of 3′3′-c-di-AMP weakens the Mtb infection. In addition, these CDNs not only stimulate dendritic cells against intracellular pathogens like Mtb but also, as adjuvants, can induce durable immune responses against intracellular pathogens like Mtb.^6,11,15,30,31^ (B) During infection, Mtb-derived CdnP is released into the host's cytosol *via* autolysis, and the Mtb CdnP degrades the host-derived 2′3′-cGAMP (STING agonist) and suppresses the host's cytosolic surveillance pathway (CSP).^11^ Inhibiting STING activity either by degrading natural or pathogen-originated STING agonists or by blocking the STING agonist binding STING receptor is a hallmark of bacterial and viral pathogens and cancer.^11,32–34^ Therefore, one strategy to combat bacterial or viral infections is to protect STING agonists from degradation by host (ectonucleotide pyrophosphatase/phosphodiesterase 1) or pathogen phosphodiesterases. Hence, blocking the Mtb CdnP catalytic activity holds great promise for the future development of therapies against infectious diseases, such as tuberculosis, by inducing innate immunity and weakening the pathogenesis factor like CdnP. FDA-approved PDE4 inhibitors (*e.g.*, roflumilast, CC-11050) are being explored as adjunctive treatments to shorten TB chemotherapy by modulating inflammation and potentially improving antibiotic penetration, suggesting that inhibiting CdnP would restore immune function against Mtb.^26,35,36^

We hypothesized that the chemically synthesized 5′-*S*-phosphorothioester linkage containing *endo-S*-2′3′-cAAMP (ES-2′3′-cAAMP) would inhibit mycobacterium CdnP.^27,37^ In the current study, we have demonstrated that ES-2′3′-cAAMP, in which the bridging oxygen and guanine base were substituted with a sulfur atom and an adenine base (Fig. 1), respectively, not only binds (Fig. 1D) to the catalytic pocket of Mtb CdnP (Fig. 4) but also effectively competes with the binding of its natural substrates, 3′3′-c-di-AMP and 3′3′-c-di-GMP, to CdnP (Fig. 2A and B), suggesting that it can be a competitive inhibitor of CdnP. Notably, ES-2′3′-cAAMP inhibits the phosphodiesterase activity of Mtb CdnP (Fig. 3B). Intriguingly, this is our first report of the Mtb CdnP complexed with the *endo-S*-CDN analog. The co-crystal structure revealed that ES-2′3′cAAMP adopted a horseshoe conformation, and this conformation is unable to bind to CdnP in a catalytically competent pose to be hydrolyzed by the enzyme (Fig. 5B). Additionally, it occupies a region of the catalytic pocket that is not accessible to the catalytic residues and the catalytic Mn^2+^ ions to cleave the phosphodiester bond of the inhibitor. Then we modelled the structure of the Mtb CdnP substrate – the CdnP–3′3′-c-di-AMP complex; as expected, c-di-AMP not only adopts an extended conformation but is also bound in the region of the catalytic pocket accessible to catalytic components for its hydrolysis by the enzyme (Fig. 7C). These comparative structural analyses, complemented by biochemical studies, have suggested that designing a new set of CDN inhibitors with a unique horseshoe-shaped conformation may improve their potency in inhibiting the phosphodiesterase activity of the CdnP enzyme. Further analyses indicate that one of the adenines of both the substrate (3′3′-c-di-AMP) and the inhibitor (ES-2′3′-cAAMP) occupies the C-site. Due to this, the substrates or CDN STING agonists (2′3′-cGAMP) are unable to bind to the catalytic pocket of CdnP (Fig. 7A and B). Interestingly, natural and modified 2′3′-cGAMP analogs adopted a horseshoe-shaped conformation in the co-crystal structures of various STING orthologs/paralogs and viral Poxin PDEs,^30,38,39^ suggesting that the 2′–5′ phosphodiester linkage in these CDNs promotes the adoption of thermodynamically stable horseshoe-shaped conformations, in comparison to both extended and horseshoe-shaped forms observed in CdnP substrates such as 3′3′-c-di-AMP,^28,40^ 3′3′-c-di-GMP,^28,41^ and 3′,3′-cGAMP.^42,43^

Similar to the bacterial pathogens Mtb, *Bacillus subtilis*, *Streptococcus pneumoniae*, and *Streptococcus pyogenes*, viruses also utilize Poxin phosphodiesterase enzymes to degrade the immune second messenger 2′3′-cGAMP, thereby suppressing cGAS–STING signaling in mammalian cells.^11,25,39,44^ Fluorinated cGAMP STING analogs that are resistant to Poxin-mediated cleavage have been explored as potential host-directed antiviral therapeutics, and arabinose or xylose sugar-derived cGAMP analogs have also been reported as STING agonists and to be resistant to degradation by the host phosphodiesterase ENPP1.^30,45^ The above cGAMP derivatives and other CDN derivatives showed enhanced stability, biocompatibility, and activity and improved pharmacological outcomes, and were thus selected for clinical advancement.^30^ Thus, ES-2′3′-cAAMP should be further evaluated as an inhibitor of the phosphodiesterases of viral and other bacterial pathogens.

## Conclusion

Overall, our study revealed that the 5′-*S*-phosphorothioester linkage containing ES-2′3′-cAAMP (A) binds to the CdnP, a phosphodiesterase of Mtb; (B) competes for substrate binding; and (C) inhibits the catalytic activity of Mtb CdnP. CdnP–ES-2′3′-cAAMP is the first representative structure of the CdnP complexed with CDN, revealing the unique pose of ES-2′3′-cAAMP in the catalytic pocket of CdnP, which prevents its degradation by the enzyme and, in parallel, obstructs substrate binding because it is bound in the catalytic pocket. Given that both ES-2′3′-cAAMP in the CdnP and 2′3′-cGAMP in the STING pocket adopt the identical conformation, ES-2′3′-cAAMP can be evaluated as a STING agonist, and thus it can be used as both a STING agonist and also an inhibitor of CdnP for two-pronged anti-TB therapy.

## Materials and methods

### Protein expression and purification

The gene encoding Mtb CdnP (UniProt ID: P71615) was cloned into the bacterial expression vector pET28-His10-Smt3 between BamHI and XhoI restriction sites, introducing an N-terminal His10-Smt3 tag. For the expression of recombinant Mtb CdnP, *E. coli* Rosetta2 DE3 cells were co-transformed with the pET28-CdnP-His10-Smt3 gene construct. A single colony was picked up and inoculated in 50 ml of LB broth, and cells were allowed to grow overnight at 37 °C, supplemented with kanamycin (50 µg ml^−1^) and chloramphenicol (35 µg ml^−1^). A 50 ml primary culture was used to inoculate a 6-liter LB secondary culture and grown at 37 °C to an OD_600_ of ∼0.6. After that, cells were induced with 0.2 mM IPTG, followed by incubation at 16 °C for 18 hours. Cells were harvested by centrifugation (Thermo Scientific Sorvall LYNX 4000) at 8000*g* for 10 min at 16 °C and further lysed in buffer containing 500 mM NaCl, 50 mM Tris-HCl (pH 8.0), 20 mM imidazole, 2 mM β-mercaptoethanol, 1 mM PMSF, and 1 mg ml^−1^ lysozyme by sonication.

After sonication, the cell lysate was clarified by centrifugation (BECKMAN COULTER AVANTI-J25I Centrifuge) at 18 000 rpm for 60 min at 4 °C. Mtb CdnP was purified by Ni-NTA affinity chromatography using a HisTrap column and eluted with buffer containing 300 mM imidazole. Eluted fractions were pooled, concentrated, and incubated with ULP1 protease (3 mg ml^−1^) at 4 °C for 4 hours to cleave the N-terminal His_10_-Smt3 (SUMO) tag from CdnP. The mixture was subjected to a second Ni-NTA affinity chromatography, and the unbound fractions containing tag-free CdnP were collected. Mtb CdnP was further purified by size-exclusion chromatography (Superdex-200-16/600, GE Healthcare) pre-equilibrated with buffer comprising 150 mM NaCl, 20 mM Tris-HCl (pH 8.0), 1 mM β-mercaptoethanol, and 5% glycerol. Eluted protein fractions were characterized by SDS-PAGE for purity. Purification of the ULP1 protease used in cleavage was performed as described before.^15^ Final protein samples were concentrated using Amicon Ultra centrifugal filters (MERCK), flash-frozen, and stored at −80 °C.

### Isothermal titration calorimetry (ITC) binding studies

ITC binding studies were performed at 20 °C using LV Affinity ITC (TA Instruments, USA). The CDNs, including 3′,3′-c-di-AMP and 3′,3′-c-di-GMP, were purchased from Sigma-Aldrich. The 5′-*S*-phosphorothioester linkage containing the 2′3′-cGAMP analogue, *i.e.*, *endo-S*-2′3′-cAAMP (abbreviated as ES-2′3′-cAAMP), in which the bridging oxygen atom of the 2′–5′ phosphodiester bond is replaced by a sulfur atom, and the guanine base is substituted with adenine, was chemically synthesized as described previously.^27^ All CDNs used in this study were dissolved in filtered, autoclaved water. For ITC binding studies, both CDNs and CdnP were dissolved in gel filtration buffer (150 mM NaCl, 20 mM Tris-HCl, pH 8.0, 1 mM β-mercaptoethanol, and 5% glycerol) without manganese salt. Individual CDN (0.3–0.5 mM) was loaded into the syringe and titrated against Mtb CdnP (10–30 µM) loaded into the cell at 19 injections, each of 2.5 µl volume, with a 3 min gap between injections at 20 °C at a stirring rate of 125 rpm. The obtained thermograms were processed using the independent binding model in NanoAnalyze software (version 3.12.5, TA Instruments, USA) to calculate the molar dissociation constant (*K*_D_), enthalpy change (Δ*H*), entropy change (Δ*S*), and binding stoichiometry (*n*) of CdnP–CDN complexes. All experiments were conducted in triplicate, and data are expressed as mean ± standard error.

### ITC competition binding studies

Competitive binding assays were performed using ITC to quantitatively assess the relative binding affinities of 3′,3′-cyclic dinucleotide substrates toward CdnP complexes pre-incubated with ES-2′3′-cAAMP. CdnP (30 µM) was pre-incubated with ES-2′3′-cAAMP at a 1 : 1 stoichiometric ratio prior to loading into the ITC sample cell. In competitive displacement experiments, 3′3′-c-di-AMP or 3′3′-c-di-GMP (1 mM) was introduced as the competing ligand *via* an injection syringe. Successive titrations (19 injections, 2.5 µl each) were carried out with the preformed CdnP–ES-2′3′-cAAMP complex in the cell, while either 3′,3′-c-di-AMP or 3′,3′-c-di-GMP was titrated from the syringe. All measurements were conducted at 20 °C with a constant stirring speed of 125 rpm to ensure homogeneous mixing and accurate heat signal acquisition. Apparent molar dissociation constants (*K*_D,apparent_) for competitive binding interactions were derived from the resulting thermograms using the independent fitting model.

### Quantification of the catalytic activity of CdnP and inhibition assays

In the presence of divalent metal ions, the Mtb CdnP hydrolyzes bis-*p*-nitrophenyl phosphate (bis-pNPP) into *p*-nitrophenol (*p*-NP),^15^ which is detectable spectrophotometrically at 410 nm wavelength. Here, bis-pNPP was used as a substrate to measure the catalytic activity and CdnP kinetics parameters. First, a standard curve for *p*-NP was generated for different concentrations of *p*-NP using a multimode plate reader (EnSpire Multimode Plate Reader, PerkinElmer, Inc.) in a 96-well plate by recording the absorbance at 410 nm. Optimal assay conditions for the Mtb CdnP catalytic activity, including pH, salt concentration, protein, and substrate concentrations, were identified as described previously.^15,46^ Afterwards, the Michaelis–Menten constant (*K*_M_), maximum velocity (*V*_max_), and turnover number (*k*_cat_) were measured by incubating 25 nM Mtb CdnP with different concentrations of bis-pNPP (ranging from 50 µM to 6.4 mM) in a 100 µl assay buffer (50 mM Tris-HCl, pH 8.0, 2.5 mM MnCl_2_, and 1 mM β-mercaptoethanol) at 37 °C for different time points. Initial rates were derived from the linear regression of progress curves and plotted against bis-pNPP concentrations for Michaelis–Menten analysis (GraphPad Prism 9.5.1). For IC_50_ determination, 25 nM Mtb CdnP was pre-incubated with varying concentrations of ES-2′3′-cAAMP (ranging from 123 nM to 2.43 mM) in a 25 µl volume at 4 °C for 30 minutes. Following ES-2′3′-cAAMP incubation, bis-pNPP (4.5 mM) was added, and the reaction was allowed proceed at 37 °C for 30 minutes before quenching with 50 mM EDTA (pH 8.0). The absorbance of liberated *p*-NP was measured at 410 nm using a multimode plate reader (EnSpire, PerkinElmer). A control reaction with 25 nM CdnP and bis-pNPP was performed under the same conditions. All assays were performed in triplicate, and enzyme activity was calculated using the *p*-NP standard curve. IC_50_ and *K*_i_ values were determined by nonlinear regression in GraphPad Prism 9.5.1, with results reported as mean ± standard error and 95% confidence intervals.

### Crystallization and data collection

The recombinantly purified Mtb CdnP was dialyzed against buffer containing 20 mM Tris-HCl (pH 8.0), 150 mM NaCl, 1 mM MnCl_2_, 1 mM βME, and 5% glycerol. For crystallization, CdnP at 10.5 mg mL^−1^ (0.3 mM) was pre-incubated with ES-2′3′-cAAMP (1 : 5 molar ratio) on ice for 30 minutes. Crystallization was performed using the hanging-drop vapor diffusion method at 20 °C in 24-well plates by mixing 1 µl of the CdnP–ES-2′3′-cAAMP complex with 1 µl of reservoir solution. Crystals appeared within 4–5 days in 0.1 M sodium acetate trihydrate (pH 4.5) and 2 M sodium formate (Fig. S1B). Single-crystal X-ray diffraction data were collected using an in-house Rigaku rotating anode X-ray source (*λ* = 1.54179 Å) at the CSIR-Centre for Cellular and Molecular Biology (CCMB), Hyderabad, India. Crystals were cryoprotected with 30% ethylene glycol prior to data collection. A total of 197 diffraction images were collected with 0.5° oscillation and a 3-minute exposure per frame. The crystal diffracted to a resolution of 2.82 Å.

### Data processing and structure determination

All the collected images were indexed and integrated using XDSGUI (version: Jan 19, 2025).^47^ Data scaling and merging were performed using the AIMLESS program of the CCP4i suite.^48^ Molecular replacement was performed using the MolRep^49^ (PMID: 20057045) of the CCP4i suite, with the ligand-free structure of Mtb CdnP as the search model (PDB ID: 5CET).^21^ RefMac5 was used for the refinement.^50^ Iterative model building was performed using WinCoot (version 0.9.8)^51^ (PMID: 20383002), and further refinements were carried out using Phenix (version 1.21).^52^ The ligand was initially sketched using ChemDraw (version 23.1)^53^ and energy-minimized using the MMFF94 force field in Avogadro (version 1.2).^54^ The ligand restraints for refinement were generated from the GRADE webserver (https://grade.globalphasing.org) before manually fitting the ligand into the unassigned electron density using Coot.^51^ The final refined model and structure factors were deposited in the RCSB Protein Data Bank under accession code 9WBU. Complete data collection and refinement statistics are presented in Table 2. Molecular graphics and structural analyses were performed using UCSF Chimera^55^ and PyMol (Schrödinger).

## Author contributions

D. S. H.: methodology, investigation, formal analysis, data curation, writing – review & editing. S. N.: methodology, investigation, data curation. K. M. S.: conceptualization, formal analysis, funding acquisition. S. K. Y.: investigation. H. O. S.: conceptualization, supervision, writing – review & editing, funding acquisition. E. R.: conceptualization, supervision, writing – review & editing, writing – original draft, project administration, funding acquisition, formal analysis.

## Conflicts of interest

There are no conflicts to declare.

## Abbreviations

CDN: Cyclic dinucleotides
CdnP: Cyclic dinucleotide phosphodiesterase
Mtb: *Mycobacterium tuberculosis*
STING: Stimulator of interferon genes
cGAMP: Cyclic GMP–AMP

## Supplementary Material

CB-007-D6CB00006A-s001

CB-007-D6CB00006A-s002

## Data Availability

Additional data supporting the findings of this study can be found in the Supplementary information (SI) of this article. Supplementary information: Fig. S1–S4. See DOI: https://doi.org/10.1039/d6cb00006a. The structural coordinates of the CdnP–ES-2′3′-cAAMP structure were deposited in the Protein Data Bank with PDB ID: 9WBU.

## References

[cit1] Gomelsky M. (2011). cAMP, c-di-GMP, c-di-AMP and now cGMP: bacteria use them all!. Mol. Microbiol..

[cit2] Kalia D., Merey G., Nakayama S., Zheng Y., Zhou J., Luo Y. (2013). *et al.*, Nucleotide, c-di-GMP, c-di-AMP, cGMP, cAMP, (p)ppGpp signaling in bacteria and implications in pathogenesis. Chem. Soc. Rev..

[cit3] Gao J., Tao J., Liang W., Zhao M., Du X., Cui S. (2015). *et al.*, Identification and characterization of phosphodiesterases that specifically degrade 3′3′-cyclic GMP-AMP. Cell Res..

[cit4] Römling U. (2008). Great times for small molecules: c-di-AMP, a second messenger candidate in Bacteria and Archaea. Sci. Signaling.

[cit5] Zaver S. A., Woodward J. J. (2020). Cyclic dinucleotides at the forefront of innate immunity. Curr. Opin. Cell Biol..

[cit6] Corrigan R. M., Gründling A. (2013). Cyclic di-AMP: another second messenger enters the fray. Nat. Rev. Microbiol..

[cit7] Dey B., Dey R. J., Cheung L. S., Pokkali S., Guo H., Lee J.-H. (2015). *et al.*, A bacterial cyclic dinucleotide activates the cytosolic surveillance pathway and mediates innate resistance to tuberculosis. Nat. Med..

[cit8] Mudgal S., Goyal N., Kasi M., Saginela R., Singhal A., Nandi S. (2024). *et al.*, Cyclic di-AMP regulates genome stability and drug resistance in Mycobacterium through RecA-dependent and RecA-independent recombination. PNAS Nexus.

[cit9] Manikandan K., Prasad D., Srivastava A., Singh N., Dabeer S., Krishnan A. (2018). *et al.*, The second messenger cyclic di-AMP negatively regulates the expression of Mycobacterium smegmatis recA and attenuates DNA strand exchange through binding to the C-terminal motif of mycobacterial RecA proteins. Mol. Microbiol..

[cit10] Su M., Zheng J., Gan L., Zhao Y., Fu Y., Chen Q. (2022). Second messenger 2′3′-cyclic GMP-AMP (2′3′-cGAMP): Synthesis, transmission, and degradation. Biochem. Pharmacol..

[cit11] Dey R. J., Dey B., Zheng Y., Cheung L. S., Zhou J., Sayre D. (2017). *et al.*, Inhibition of innate immune cytosolic surveillance by an *M. tuberculosis* phosphodiesterase. Nat. Chem. Biol..

[cit12] Wright T. A., Jiang L., Park J. J., Anderson W. A., Chen G., Hallberg Z. F. (2020). *et al.*, Second messengers and divergent HD-GYP phosphodiesterases regulate 3′,3′-cGAMP signaling. Mol. Microbiol..

[cit13] Opoku-Temeng C., Zhou J., Zheng Y., Su J., Sintim H. O. (2016). Cyclic dinucleotide (c-di-GMP, c-di-AMP, and cGAMP) signalings have come of age to be inhibited by small molecules. Chem. Commun..

[cit14] Yin W., Cai X., Ma H., Zhu L., Zhang Y., Chou S.-H. (2020). *et al.*, A decade of research on the second messenger c-di-AMP. FEMS Microbiol. Rev..

[cit15] Manikandan K., Sabareesh V., Singh N., Saigal K., Mechold U., Sinha K. M. (2014). Two-step synthesis and hydrolysis of cyclic di-AMP in Mycobacterium tuberculosis. PLoS One.

[cit16] da Purificação A. D., de Azevedo N. M., de Araujo G. G., de Souza R. F., Guzzo C. R. (2020). The World of Cyclic Dinucleotides in Bacterial Behavior. Molecules.

[cit17] Louie A., Bhandula V., Portnoy D. A. (2020). Secretion of c-di-AMP by *Listeria monocytogenes* Leads to a STING-Dependent Antibacterial Response during Enterocolitis. Infect. Immun..

[cit18] Schoggins J. W., Wilson S. J., Panis M., Murphy M. Y., Jones C. T., Bieniasz P. (2011). *et al.*, A diverse range of gene products are effectors of the type I interferon antiviral response. Nature.

[cit19] Whiteley A. T., Garelis N. E., Peterson B. N., Choi P. H., Tong L., Woodward J. J. (2017). *et al.*, c-di-AMP modulates *Listeria monocytogenes* central metabolism to regulate growth, antibiotic resistance and osmoregulation. Mol. Microbiol..

[cit20] Sun Y., Zhang W., Dong C., Xiong S. (2020). Mycobacterium tuberculosis MmsA (Rv0753c) Interacts with STING and Blunts the Type I Interferon Response. mBio.

[cit21] He Q., Wang F., Liu S., Zhu D., Cong H., Gao F. (2016). *et al.*, Structural and Biochemical Insight into the Mechanism of Rv2837c from Mycobacterium tuberculosis as a c-di-NMP Phosphodiesterase. J. Biol. Chem..

[cit22] Bai Y., Yang J., Eisele L. E., Underwood A. J., Koestler B. J., Waters C. M. (2013). *et al.*, Two DHH subfamily 1 proteins in Streptococcus pneumoniae possess cyclic di-AMP phosphodiesterase activity and affect bacterial growth and virulence. J. Bacteriol..

[cit23] Cho K. H., Kang S. O. (2013). Streptococcus pyogenes c-di-AMP phosphodiesterase, GdpP, influences SpeB processing and virulence. PLoS One.

[cit24] Du B., Ji W., An H., Shi Y., Huang Q., Cheng Y. (2014). *et al.*, Functional analysis of c-di-AMP phosphodiesterase, GdpP, in Streptococcus suis serotype 2. Microbiol. Res..

[cit25] Rao F., See R. Y., Zhang D., Toh D. C., Ji Q., Liang Z.-X. (2010). YybT is a signaling protein that contains a cyclic dinucleotide phosphodiesterase domain and a GGDEF domain with ATPase activity. J. Biol. Chem..

[cit26] Ahidjo B. A., Bishai W. R. (2016). Phosphodiesterase inhibitors as adjunctive therapies for tuberculosis. EBioMedicine.

[cit27] Yeboah S. K., Meher S., Harper H. A., McMahan C., Elzey B. D., Sintim H. O. (2025). 5′-Phosphorothioester Linked Cyclic Dinucleotides, *Endo-S*-CDNs, Displaying Impressive Antitumor Activities In Vivo when Dosed Subcutaneously. ACS Bio Med Chem Au.

[cit28] Wang F., He Q., Su K., Wei T., Xu S., Gu L. (2018). Structural and biochemical characterization of the catalytic domains of GdpP reveals a unified hydrolysis mechanism for the DHH/DHHA1 phosphodiesterase. Biochem. J..

[cit29] Drexler D. J., Müller M., Rojas-Cordova C. A., Bandera A. M., Witte G. (2017). Structural and Biophysical Analysis of the Soluble DHH/DHHA1-Type Phosphodiesterase TM1595 from Thermotoga maritima. Structure.

[cit30] Klima M., Dejmek M., Duchoslav V., Eisenreichova A., Sala M., Chalupsky K. (2024). *et al.*, Fluorinated cGAMP analogs, which act as STING agonists and are not cleavable by poxins: Structural basis of their function. Structure.

[cit31] Huynh T. N., Luo S., Pensinger D., Sauer J.-D., Tong L., Woodward J. J. (2015). An HD-domain phosphodiesterase mediates cooperative hydrolysis of c-di-AMP to affect bacterial growth and virulence. Proc. Natl. Acad. Sci. U. S. A..

[cit32] Fu Y.-Z., Su S., Gao Y.-Q., Wang P.-P., Huang Z.-F., Hu M.-M. (2017). *et al.*, Human
Cytomegalovirus Tegument Protein UL82 Inhibits STING-Mediated Signaling to Evade Antiviral Immunity. Cell Host Microbe.

[cit33] Wang A., Peng Q., Fan H., Ji W., Lou J., Zhou X. (2025). *et al.*, Herpes simplex virus 1 encodes a STING antagonist that can be therapeutically targeted. Cell Rep. Med..

[cit34] Zheng Z.-Q., Fu Y.-Z., Wang S.-Y., Xu Z.-S., Zou H.-M., Wang Y.-Y. (2022). Herpes simplex virus protein UL56 inhibits cGAS-Mediated DNA sensing to evade antiviral immunity. Cell Insight.

[cit35] Maiga M. C., Ahidjo B. A., Maiga M., Bishai W. R. (2015). Roflumilast, a Type 4 Phosphodiesterase Inhibitor, Shows Promising Adjunctive, Host-Directed Therapeutic Activity in a Mouse Model of Tuberculosis. Antimicrob. Agents Chemother..

[cit36] Subbian S., Tsenova L., Holloway J., Peixoto B., O’Brien P., Dartois V. (2016). *et al.*, Adjunctive Phosphodiesterase-4 Inhibitor Therapy Improves Antibiotic Response to Pulmonary Tuberculosis in a Rabbit Model. EBioMedicine.

[cit37] Yeboah S. K., Sintim H. O. (2024). PDE-stable 2′3′-cGAMP analogues, containing 5′-S-phosphorothioester linkage, as STING agonists. RSC Med. Chem..

[cit38] Zhang X., Shi H., Wu J., Zhang X., Sun L., Chen C. (2013). *et al.*, Cyclic GMP-AMP containing mixed phosphodiester linkages is an endogenous high-affinity ligand for STING. Mol. Cell.

[cit39] Eaglesham J. B., Pan Y., Kupper T. S., Kranzusch P. J. (2019). Viral and metazoan poxins are cGAMP-specific nucleases that restrict cGAS-STING signalling. Nature.

[cit40] Chin K.-H., Liang J.-M., Yang J.-G., Shih M.-S., Tu Z.-L., Wang Y.-C. (2015). *et al.*, Structural Insights into the Distinct Binding Mode of Cyclic Di-AMP with SaCpaA_RCK. Biochemistry.

[cit41] Benach J., Swaminathan S. S., Tamayo R., Handelman S. K., Folta-Stogniew E., Ramos J. E. (2007). *et al.*, The structural basis of cyclic diguanylate signal transduction by PilZ domains. EMBO J..

[cit42] Ren A., Wang X. C., Kellenberger C. A., Rajashankar K. R., Jones R. A., Hammond M. C. (2015). *et al.*, Structural basis for molecular discrimination by a 3′,3′-cGAMP sensing riboswitch. Cell Rep..

[cit43] Yadav M., Pal K., Sen U. (2019). Structures of c-di-GMP/cGAMP degrading phosphodiesterase VcEAL: identification of a novel conformational switch and its implication. Biochem. J..

[cit44] Srivastav R., Kumar D., Grover A., Singh A., Manjasetty B. A., Sharma R. (2014). *et al.*, Unique subunit packing in mycobacterial nanoRNase leads to alternate substrate recognitions in DHH phosphodiesterases. Nucleic Acids Res..

[cit45] Xie W., Lama L., Yang X., Kuryavyi V., Bhattacharya S., Nudelman I. (2023). *et al.*, Arabinose- and xylose-modified analogs of 2′,3′-cGAMP act as STING agonists. Cell. Chem. Biol..

[cit46] Smirnovienė J., Baranauskienė L., Zubrienė A., Matulis D. (2021). A standard operating procedure for an enzymatic activity inhibition assay. Eur. Biophys. J..

[cit47] Kabsch W. (2010). XDS. Acta Crystallogr., Sect. D:Biol. Crystallogr..

[cit48] Evans P. R., Murshudov G. N. (2013). How good are my data and what is the resolution?. Acta Crystallogr., Sect. D:Biol. Crystallogr..

[cit49] Vagin A., Teplyakov A. (2010). Molecular replacement with MOLREP. Acta Crystallogr., Sect. D:Biol. Crystallogr..

[cit50] Murshudov G. N., Skubák P., Lebedev A. A., Pannu N. S., Steiner R. A., Nicholls R. A. (2011). *et al.*, REFMAC5 for the refinement of macromolecular crystal structures. Acta Crystallogr., Sect. D:Biol. Crystallogr..

[cit51] Emsley P., Lohkamp B., Scott W. G., Cowtan K. (2010). Features and development of Coot. Acta Crystallogr., Sect. D:Biol. Crystallogr..

[cit52] Adams P. D., Afonine P. V., Bunkóczi G., Chen V. B., Davis I. W., Echols N. (2010). *et al.*, PHENIX: a comprehensive Python-based system for macromolecular structure solution. Acta Crystallogr., Sect. D:Biol. Crystallogr..

[cit53] Li Z., Wan H., Shi Y., Ouyang P. (2004). Personal experience with four kinds of chemical structure drawing software: review on ChemDraw, ChemWindow, ISIS/Draw, and ChemSketch. J. Chem. Inf. Comput. Sci..

[cit54] Hanwell M. D., Curtis D. E., Lonie D. C., Vandermeersch T., Zurek E., Hutchison G. R. (2012). Avogadro: an advanced semantic chemical editor, visualization, and analysis platform. J. Cheminf..

[cit55] Pettersen E. F., Goddard T. D., Huang C. C., Couch G. S., Greenblatt D. M., Meng E. C. (2004). *et al.*, UCSF Chimera–a visualization system for exploratory research and analysis. J. Comput. Chem..

