# Supplementary material for: Structural and biochemical insights into the inhibition of Mycobacterium tuberculosis cyclic dinucleotide phosphodiesterase by a sulfur-modified cyclic dinucleotide analog
Source: RSC Chem Biol. 2026 Apr 14;7(5):880–91. doi: 10.1039/d6cb00006a (PMC13077336; doi:10.1039/d6cb00006a)
Supplement: CB-007-D6CB00006A-s002 [file CB-007-D6CB00006A-s002.pdf]

Supplementary Information (SI) for RSC Chemical Biology.
This journal is © The Royal Society of Chemistry 2026

# Supplementary Information

## Structural and biochemical insights into the inhibition of *Mycobacterium tuberculosis* Cyclic Dinucleotide Phosphodiesterase by a sulfur-modified cyclic dinucleotide analog

Dagur Singh Hanuman[1,$], Singh Neeharika[1,$], Sinha Krishna Murari[2], Simpa K. Yeboah[3], Herman O. Sintim[#,3,4], and Eerappa Rajakumara[1,*]

[1]Macromolecular Structural Biology Laboratory, Department of Biotechnology, Indian Institute of Technology Hyderabad (IITH), Hyderabad, Telangana, India- 502285

[2]Amity Institute of Biotechnology, Amity University Haryana, Haryana, India

[3]James Tarpo Jr. and Margaret Tarpo Department of Chemistry, 560 Oval Drive, West Lafayette, IN 47907, USA.

[4]Department of Chemistry and Biochemistry, University of Notre Dame, 305A McCourtney Hall, Notre Dame, IN 46556, USA.

[$]Equally contributed

[*]Corresponding author: Eerappa Rajakumara; Email: eraj@bt.iith.ac.in

[#]Co-corresponding author: Herman O. Sintim, Email: hsintim@nd.edu

**Supplementary Figures**

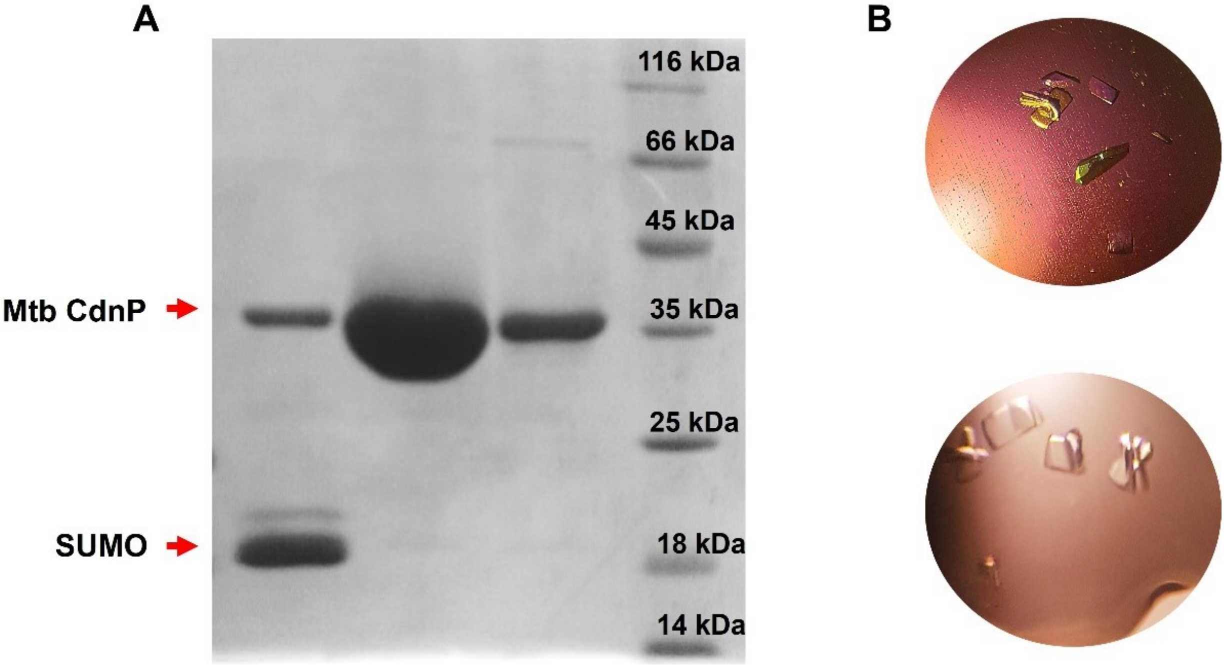


**Figure S1. Purification and crystallization of M. tuberculosis CdnP.** (A) SDS-PAGE analysis of gel filtration–purified recombinant CdnP. (B) Crystal of the CdnP–ES-2'3'–cAAMP complex grown at 20 °C.

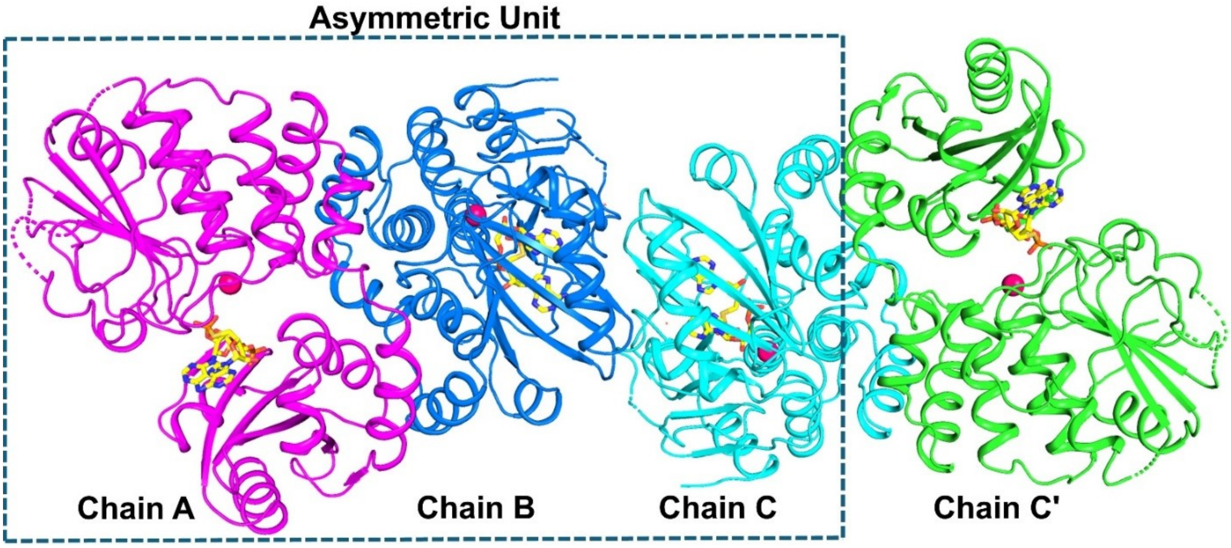


**Figure S2. Biological assembly of CdnP.** The functional dimer of CdnP is formed in two distinct ways: one dimer is assembled from two protomers (chains A and B) within the

asymmetric unit, while a second dimer is formed by chain C and its crystallographic symmetry mate (chain C′).

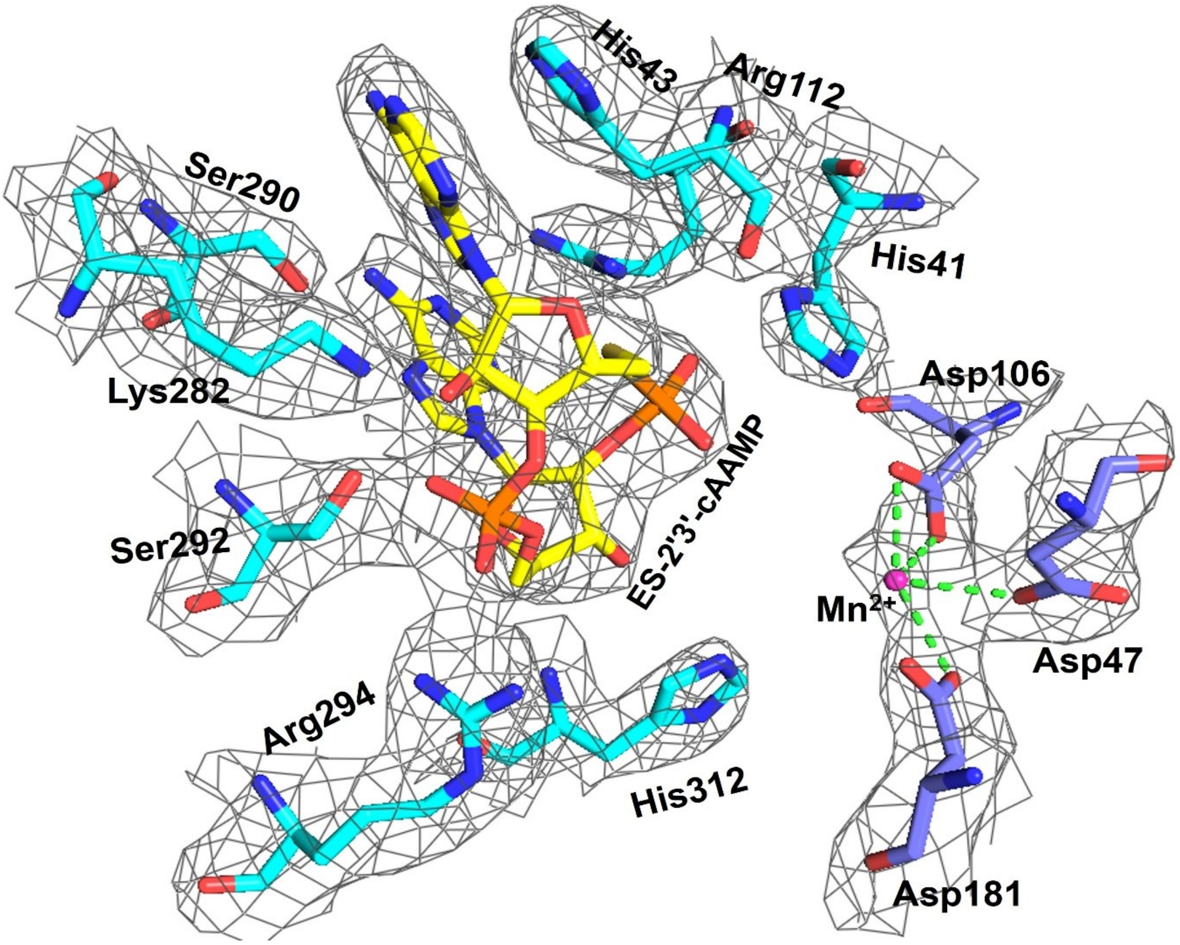


**Figure S3. Electron density map of the $Mn^{2+}$ ion and active-site residues.** The 2Fo–Fc electron density map (grey mesh, contoured at 1.0 σ) is shown around the $Mn^{2+}$ ion, catalytic Asp residue, and ES-2'3'-cAAMP-interacting residues corresponding to those shown in Fig. 5. The $Mn^{2+}$-coordinating residues are shown as light blue sticks, ES-2'3'-cAAMP-interacting residues as cyan sticks, and ES-2'3'-cAAMP as yellow sticks. $Mn^{2+}$ coordination is indicated by green dashed lines. The stacking interaction is omitted for clarity.

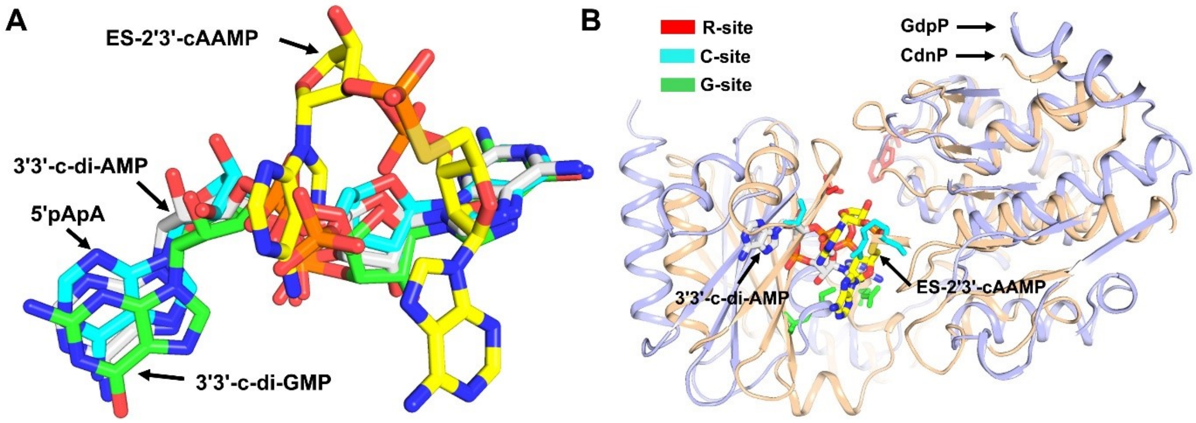


**Figure S4. Distinct CDN Binding Conformations and Site Preferences in CdnP and GdpP.** (A) The ES-2'3'–cAAMP bound to CdnP was superimposed with CDNs bound to GdpP. The 3'3'-c-di-AMP (PDB ID: 5XSN), 3'3'-c-di-GMP (PDB ID: 5XT3) and 5'pApA (PDB ID: 5XSP) exist in the extended conformations, while the ES-2'3'–cAAMP has a horseshoe-shape conformation. (B) 3′3′-c-di-AMP binds to GdpP at the GC site, whereas in CdnP it occupies the RC site. In contrast, ES-2'3'cAAMP binds to CdnP at the GC site. The R site in GdpP is not sufficiently spacious to accommodate a nucleoside moiety. R, C and G sites correspond to CdnP.
